# Age-Specific Associations of Renal Impairment With Magnetic Resonance Imaging Markers of Cerebral Small Vessel Disease in Transient Ischemic Attack and Stroke

**DOI:** 10.1161/STROKEAHA.117.019650

**Published:** 2018-03-26

**Authors:** Bian Liu, Kui Kai Lau, Linxin Li, Caroline Lovelock, Ming Liu, Wilhelm Kuker, Peter M. Rothwell

**Affiliations:** From the Centre for Prevention of Stroke and Dementia, Nuffield Department of Clinical Neurosciences University of Oxford, United Kingdom (B.L., K.K.L., L.L., C.L., W.K., P.M.R.); and Department of Neurology, Cerebrovascular Centre, West China Hospital, Sichuan University (B.L., M.L.).

**Keywords:** cerebral small vessel disease, chronic kidney disease, magnetic resonance imaging, stroke, transient ischemic attack

## Abstract

**Background and Purpose—:**

It has been hypothesized that cerebral small vessel disease (SVD) and chronic renal impairment may be part of a multisystem small-vessel disorder, but their association may simply be as a result of shared risk factors (eg, hypertension) rather than to a systemic susceptibility to premature SVD. However, most previous studies were hospital based, most had inadequate adjustment for hypertension, many were confined to patients with lacunar stroke, and none stratified by age.

**Methods—:**

In a population-based study of transient ischemic attack and ischemic stroke (OXVASC [Oxford Vascular Study]), we evaluated the magnetic resonance imaging markers of cerebral SVD, including lacunes, white matter hyperintensities, cerebral microbleeds, and enlarged perivascular space. We studied the age-specific associations of renal impairment (estimated glomerular filtration rate <60 mL/min per 1.73 m^2^) and total SVD burden (total SVD score) adjusting for age, sex, vascular risk factors, and premorbid blood pressure (mean blood pressure during 15 years preevent).

**Results—:**

Of 1080 consecutive patients, 1028 (95.2%) had complete magnetic resonance imaging protocol and creatinine measured at baseline. Renal impairment was associated with total SVD score (odds ratio [OR], 2.16; 95% confidence interval [CI], 1.69–2.75; *P*<0.001), but only at age <60 years (<60 years: OR, 3.97; 95% CI, 1.69–9.32; *P*=0.002; 60–79 years: OR, 1.01; 95% CI, 0.72–1.41; *P*=0.963; ≥80 years: OR, 0.95; 95% CI, 0.59–1.54; *P*=0.832). The overall association of renal impairment and total SVD score was also attenuated after adjustment for age, sex, history of hypertension, diabetes mellitus, and premorbid average systolic blood pressure (adjusted OR, 0.76; 95% CI, 0.56–1.02; *P*=0.067), but the independent association of renal impairment and total SVD score at age <60 years was maintained (adjusted OR, 3.11; 95% CI, 1.21–7.98; *P*=0.018). Associations of renal impairment and SVD were consistent for each SVD marker at age <60 years but were strongest for cerebral microbleeds (OR, 5.84; 95% CI, 1.45–23.53; *P*=0.013) and moderate–severe periventricular white matter hyperintensities (OR, 6.28; 95% CI, 1.54–25.63; *P*=0.010).

**Conclusions—:**

The association of renal impairment and cerebral SVD was attenuated with adjustment for shared risk factors at older ages, but remained at younger ages, consistent with a shared susceptibility to premature disease.

## Abstract 2

Supplemental Digital Content is available in the text.

It has been hypothesized that cerebral small vessel disease (SVD) may be part of a multisystem disorder affecting other vascular beds, such as the kidney.^1,2^ However, previous studies of the associations of chronic renal impairment and imaging makers of SVD were conflicting.^3–8^ While some studies suggested that the association of renal impairment and SVD was explained by shared risk factors such as hypertension, others proposed that genetic factors might contribute to shared susceptibility. Any association caused by shared genetic susceptibility would usually be strongest at younger ages and should remain even after detailed adjustment for shared vascular risk factors.

Most previous studies of the association of renal impairment and SVD were hospital based, many had small numbers, the majority were confined to lacunar stroke patients, and all previous studies only adjusted for history of hypertension or a single recent blood pressure, which does not allow adequate adjustment for the potential confounding by long-term blood pressure burden. Moreover, few studies stratified the analyses by age, and no study has focused on the associations of renal impairment and cerebral SVD at younger ages.^7–10^

Therefore, in a population-based study, the OXVASC (Oxford Vascular Study), we studied patients with transient ischemic attack (TIA) and minor ischemic stroke to determine the age-specific associations of renal impairment and the overall burden of SVD (total SVD score),^9^ as well as individual SVD markers, with adjustment for hypertension based on the average premorbid blood pressure level over many years, and by using both the premorbid and baseline creatinine measurement for the diagnosis of renal impairment.

## Methods

Requests for access to data from OXVASC will be considered by the corresponding author.

We studied consecutive patients with TIA or ischemic stroke who underwent cerebral magnetic resonance imaging in OXVASC from 2004 to 2014. OXVASC is an ongoing population-based study of the incidence and outcome of all acute vascular events in a population of 92 728 individuals, registered with 100 general practitioners in 9 general practices in Oxfordshire, United Kingdom. The multiple overlapping methods used to achieve near complete ascertainment of all individuals with TIA and ischemic stroke and the imaging protocol of OXVASC are detailed in Methods in the online-only Data Supplement and have been reported previously.^11,12^ All cases were reviewed by the senior study neurologist (Dr Rothwell), and TIA/stroke etiology was classified according to the modified Trial of ORG 10172 in Acute Stroke Treatment criteria.^11^ For the current analyses, patients with cerebral or systemic vasculitis, Cerebral Autosomal Dominant Arteriopathy With Subcortical Infarcts and Leukoencephalopathy, or Fabry disease were excluded.

Demographic data, vascular risk factors (hypertension, diabetes mellitus, known atrial fibrillation, history of smoking, hyperlipidemia), history of previous TIA/stroke, and history of ischemic heart disease were collected from face-to-face interview and cross-referenced with primary care records. Patients routinely had creatinine measured after the acute event as part of the standard protocol. We also collected all premorbid blood pressure readings with dates up to at least 15 years before the event from patient records held in primary care. The most recent premorbid creatinine measurement within 1 year of the index event was also obtained from the regional biochemistry database.

The MDRD (Modification of Diet in Renal Disease) Study Group equation was used to calculate estimated glomerular filtration rate (eGFR) for each patient, and renal impairment was defined as estimated eGFR <60 mL/min per 1.73 m^2^.^13^ To minimize the potential impact of acute renal injury after TIA and ischemic stroke, we used creatinine taken at the time of the index event in the primary analysis, and the most recent premorbid creatinine taken within 1 year of the index event was also used for sensitivity analysis.

One neuroradiologist (Dr Kuker) provided ongoing supervision of interpretation of the magnetic resonance images throughout the study period, and the independently derived and proposed total SVD score was used to assess the overall burden of SVD.^9^ One point is allocated to each of the following: (1) presence of lacunes; (2) presence of cerebral microbleeds (CMB); (3) moderate–severe (>10) basal ganglia (BG) perivascular spaces (PVS), and (4) severe periventricular or moderate–severe deep white matter hyperintensity (WMH). Lacunes were defined as rounded or ovoid lesions, >3 and <20 mm in diameter, in the BG, internal capsule, centrum semiovale, or brain stem, of cerebrospinal fluid signal density on T2 and fluid-attenuated inversion recovery and no increased signal on diffusion-weighted imaging.^14^ CMBs were defined as rounded, hypodense foci up to 10 mm in size and were differentiated from microbleed mimics based on current guidelines.^15^ PVSs were defined as small (<3 mm) punctate (if perpendicular to the plane of scan) or linear (if longitudinal to the plane of scan) hyperintensities on T2 images in BG or centrum semiovale based on a previously validated scale,^16^ and only BG-PVS were used in the total SVD score. The severity of white matter disease was determined for periventricular versus deep WMH, respectively, according to the Fazekas scale.^17^

### Statistical Analysis

Categorical variables are reported as absolute numbers with percentages, and continuous variables are reported as means with SD. χ^2^ and analysis of variance tests were performed to compare categorical and continuous variables between groups.

We first used ordinal regression to determine the age-specific (overall/stratified by age groups: <60, 60–79, and ≥80 years) associations of renal impairment and the total SVD score. We then used logistic regression to study the age-specific associations of renal impairment and individual SVD markers, including presence of lacunes, presence of CMBs, BG-PVS, moderate–severe periventricular WMH, and moderate–severe deep WMH. All analyses were adjusted for age (continuous/per year), sex, history of hypertension, diabetes mellitus, and 15-year premorbid systolic blood pressure.

All analyses were performed using SPSS version 20 (SPSS Inc, Chicago, IL).

### Standard Protocol Approvals, Registrations, and Patient Consents

Written informed consent or assent from relatives was obtained for all participants. OXVASC was approved by the local research ethics committee (OREC A: 05/Q1604/70).

## Results

Among 1080 consecutive patients with TIA or ischemic stroke who underwent magnetic resonance brain imaging, 1028 (95.2%) had a full magnetic resonance imaging protocol completed and creatinine measured at baseline and were, thus, included in the analyses. Mean (SD) age was 68.4 (14.1) years, and 261 (26.7%) were <60 years. The baseline characteristics of patients stratified by the total SVD score are shown in Table 1.

**Table 1.**
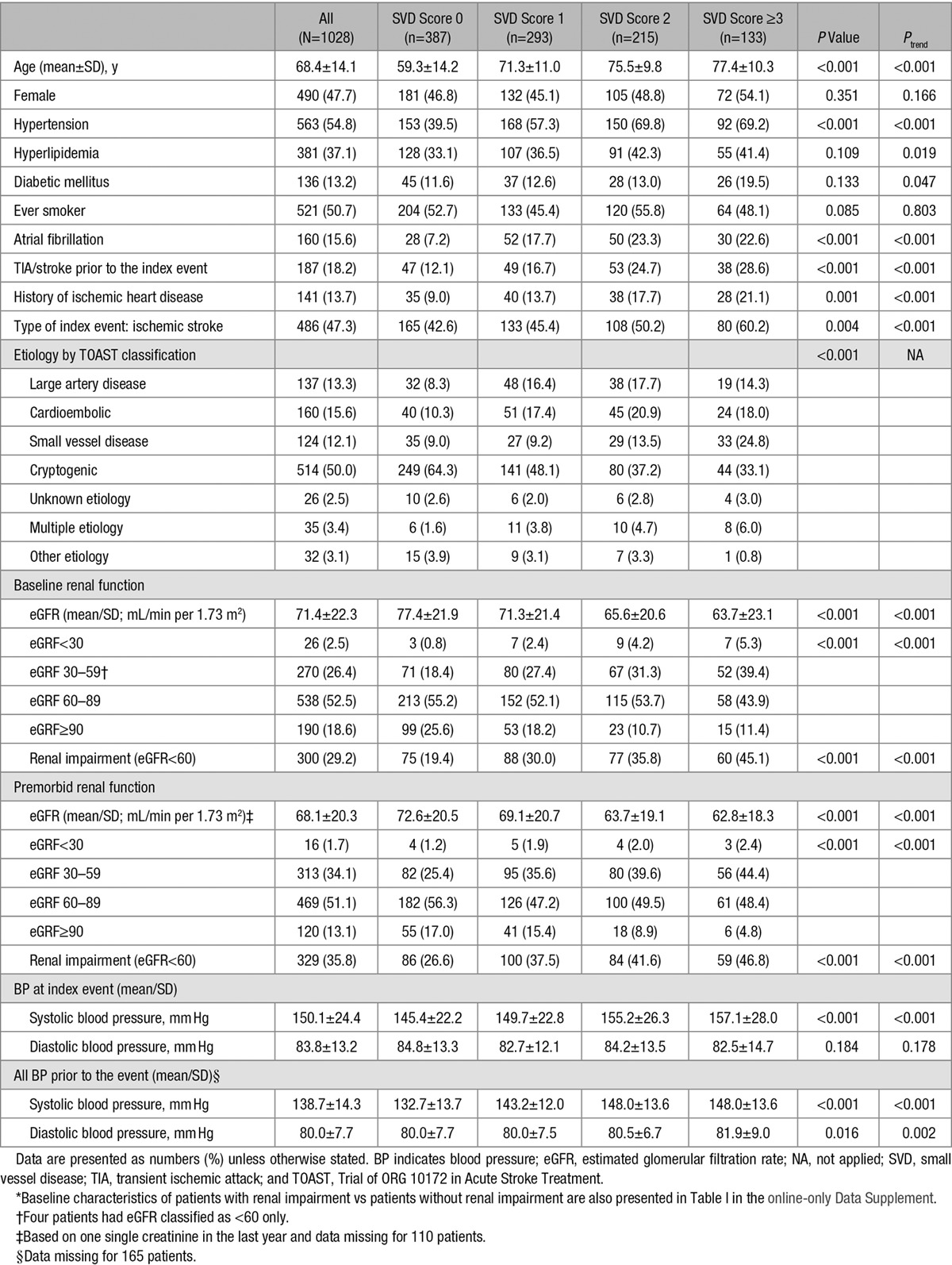
Baseline Characteristics of Patients Included in Analyses Stratified by the Total SVD Score*

As expected, patients with higher total SVD score were older, more likely to have history of hypertension, atrial fibrillation, TIA/stroke prior to the index event and history of ischemic heart disease (Table 1), and had higher blood pressure measured both acutely and during the 15 years before the index event (Table 1).

The eGFR decreased with increasing total SVD score using creatinine measured either at the index event or 1 year before the index event (both *P*_trend_<0.001; Table 1). In an ordinal regression, renal impairment (eGFR<60 mL/min per 1.73 m^2^) was associated with total SVD score (odds ratio [OR], 2.16, 95% confidence interval [CI], 1.69–2.75; *P*<0.001; Table 2), but this association was only apparent at age <60 years (<60 years: OR, 3.97; 95% CI, 1.69–9.32; *P*=0.002; 60–79 years: OR, 1.01; 95% CI, 0.72–1.41; *P*=0.963; ≥80 years: OR, 0.95; 95% CI, 0.59–1.54; *P*=0.832). The overall association of renal impairment and total SVD score was lost after adjustment for age and sex (OR, 0.94; 95% CI, 0.72–1.23; *P*=0.639; Table 2), and with additional adjustment for history of hypertension (OR, 0.85; 95% CI, 0.65–1.12; *P*=0.247; Table 2), and tended to be reversed when also adjusting for premorbid average systolic blood pressure (OR, 0.76; 95% CI, 0.56–1.02; *P*=0.067; Table 2). However, although the similar attenuation was observed for all age groups, the independent association of renal impairment and total SVD score was maintained in the multivariate analyses at age <60 years (adjusted OR, 3.11; 95% CI, 1.21–7.98; *P*=0.018; Table 2). Results were similar in patients with lacunar events and in those with nonlacunar events (Table II in the online-only Data Supplement).

**Table 2.**
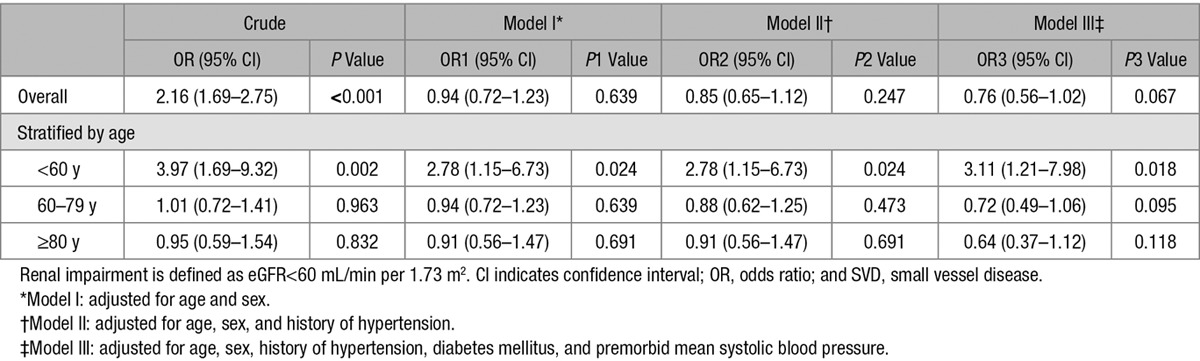
Associations of Renal Impairment and Total SVD Score Stratified by Age and Adjusted for Age/Sex and for Known Vascular Risk Factors

When we looked at the associations of renal impairment and individual SVD markers, the results were also consistent, with attenuation of apparent associations after adjustment for known risk factors but with independent associations of renal impairment and SVD remaining at younger ages (Figure and Table III in the online-only Data Supplement), particularly for the presence of CMB (OR, 5.84; 95% CI, 1.45–23.53; *P*=0.013; Figure; Table III in the online-only Data Supplement) and for moderate–severe periventricular WMH (OR, 6.28; 95% CI, 1.54–25.63; *P*=0.010; Figure; Table III in the online-only Data Supplement).

**Figure.**
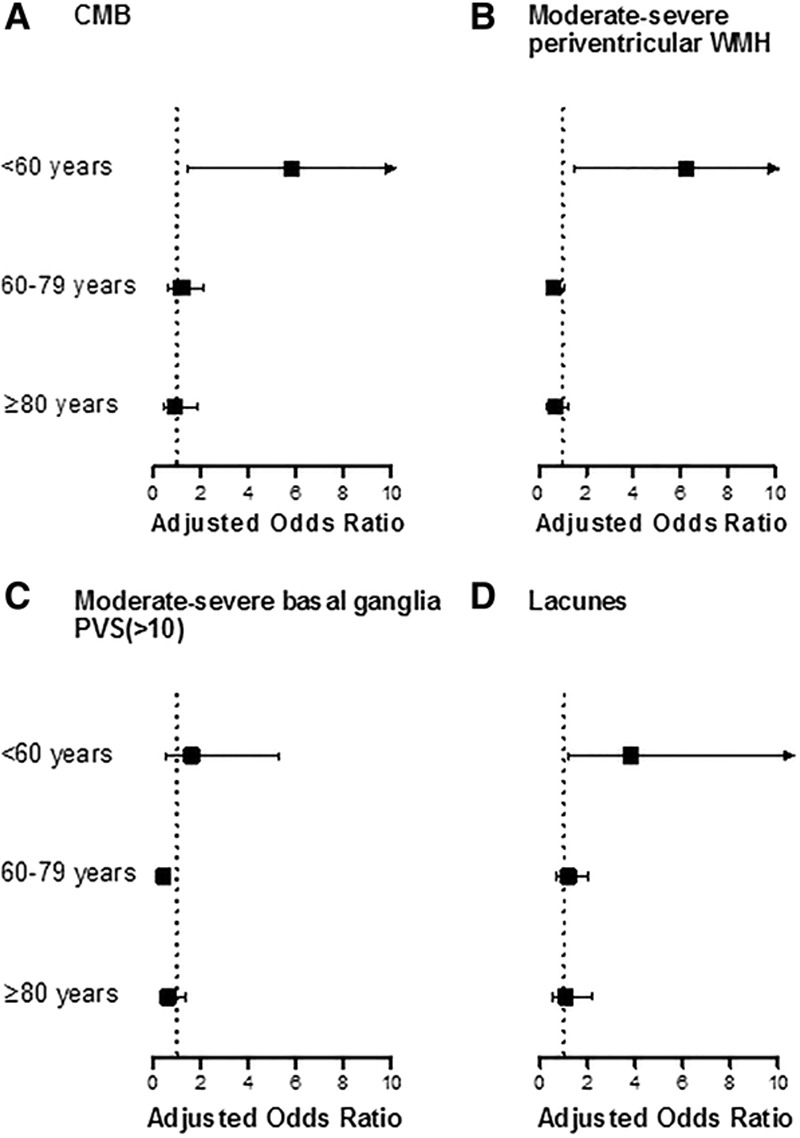
Associations of renal impairment (adjusted odds ratio and 95% CI) and the presence of individual small vessel disease markers stratified by age. Analyses were adjusted for age, sex, history of hypertension, diabetes mellitus, and premorbid mean systolic blood pressure. Renal impairment is defined as eGFR<60 mL/min per 1.73 m^2^. CI indicates confidence interval; CMB, cerebral microbleeds; eGFR, estimated glomerular filtration rate; PVS, perivascular spaces; and WMH, white matter hyperintensity.

Sensitivity analyses using creatinine measured 1 year prior to the index event also showed consistent results (Table IV and V in the online-only Data Supplement).

## Discussion

In this population-based study of patients with TIA and ischemic stroke, we found that the associations of renal impairment (eGFR<60 mL/min per 1.73 m^2^) and SVD were attenuated after adjustment for age, sex, known risk factors, and premorbid average blood pressure and disappeared at older ages. However, the association was maintained at age <60 years, both for the overall SVD burden and for individual SVD markers.

Our findings of age-specific associations of renal impairment and cerebral SVD in TIA and minor stroke are in line with previous studies done predominantly in lacunar stroke or in the nonstroke population using individual SVD markers. A rigorous and comprehensive systematic review and meta-analysis showed that the 4-fold increased risk of renal impairment in lacunar versus nonlacunar stroke was only observed at younger ages.^8^ Similarly, in the nonstroke population, compared with studies of a mean age of 70 years, there was a stronger relationship between renal impairment and WMH in studies with an average age of 50 to 60 years.^8^ The same pattern was also seen for renal impairment and CMB or enlarged PVS, where studies including younger patients tended to report a strong association^5,7^ and studies of older cohorts tended to find no association.^4^ However, one hospital-based study in TIA and ischemic stroke of a similar mean age to OXVASC (70 years versus 68.4 years) reported a strong association of proteinuria and CMB,^18^ but only adjusted for history of diagnosed hypertension.

The reason why renal impairment correlates with SVD independent of hypertension and other vascular risk factors at younger ages is uncertain. One explanation is that rather than being the end organ damage from vascular risk factors such as hypertension in 2 different systems, renal impairment and cerebral SVD could be part of a multisystem disease directly affecting small vessels more generally. Our findings that the independent association of renal impairment and SVD seemed to be strongest in those presenting with acute small vessel disease (ie, acute lacunar event) also supported this hypothesis and are consistent with the previous systematic review of renal impairment and lacunar stroke.^8^ Multisystem pathogenesis is also supported by associations of cerebral SVD with transforming growth factor-β signaling, which has also been associated with cancer, inflammation, and autoimmune diseases.^19,20^ Alternatively, the independent associations of renal impairment and SVD at younger ages could similarly suggest shared susceptibility to vascular risk factors, most likely at the genetic level, leading to premature disease.

Notably, we did not observe any apparent associations of renal impairment and SVD at older ages. Moreover, after adjustment for age, sex, vascular risk factors, and premorbid blood pressure level, the association of renal impairment and SVD even showed a trend of reversed relationship at older ages. Given that both renal impairment and SVD are associated with premature death,^21^ the nonassociation of renal impairment and SVD could be explained by a survival bias at older ages, where patients with stronger associations of renal impairment and SVD might have died at a younger age and were not therefore “available” to be recruited into the study. Similarly, patients with multiple comorbidities might also die prematurely, leaving those with fewer risk factors or less susceptibility to risk factors in the cohort, leading to a reverse association after adjustment for these risk factors.

A strength of our study is that we were able to adjust associations for long-term premorbid mean blood pressure, but there are also limitations. First, we used the creatinine-based MDRD calculation for eGFR. The MDRD equation was derived from a population with a mean age of 50.6±12.7 years.^22^ Therefore, the eGFR calculation might not be sensitive enough to differentiate between normal aging-related renal impairment versus pathological renal impairment at older ages. However, the current clinical diagnosis of renal impairment is based on the same eGFR cutoff irrespective of age. Second, we did not measure cystatin C, which may be a more sensitive marker when the creatinine-based eGFR is between 45 and 59 mL/min per 1.73 m^2^.^13^ Therefore, we might have overestimated the true prevalence of renal impairment. Even so, we still found an independent association of renal impairment and SVD at younger ages. Third, we did not have data on proteinuria and used eGFR measurement after the acute event for the diagnosis of renal impairment. However, our sensitivity analyses using the eGFR prior to the index event showed consistent results. Fourth, we used the total SVD score to assess the burden of cerebral SVD. However, quantitative measurements of SVD markers might be more accurate in measuring the overall burden particularly for the more severe end. Fifth, although we adjusted for known confounding factors, the possibility of residual confounding could not be excluded. Moreover, we did not adjust for long-term blood pressure variability, although preliminary analyses do not suggest any significant confounding. Sixth, the multiple subgroup analyses were mainly for hypothesis generating and should therefore be interpreted with caution. Finally, our study is based in a predominantly White population and might not be generalizable to the Asian population, where there seems to be stronger association of renal impairment and SVD.

Our study has several implications. First, the independent associations of renal impairment and SVD at younger ages highlight the importance of effective renal function monitoring and management for young patients. Second, young patients with renal impairment and SVD could be potentially an interesting group for future genetic studies of small vessel disease, and future studies should stratify analyses by age. Finally, further research is needed to understand if there is age-specific treatment effect of renal impairment management on reducing the overall burden of cerebral SVD.

## Acknowledgments

We are grateful to all the staff in the general practices that collaborated in the Oxford Vascular Study: Abingdon Surgery, Stert St, Abingdon; Malthouse Surgery, Abingdon; Marcham Road Family Health Centre, Abingdon; The Health Centre, Berinsfield; Key Medical Practice; Kidlington; 19 Beaumont St, Oxford; East Oxford Health Centre, Oxford; Church Street Practice, Wantage. We also acknowledge the use of the facilities of the Acute Vascular Imaging Centre, Oxford. Dr B Liu collected data, did the statistical analysis and interpretation, wrote, and revised the manuscript. Dr KK Lau, Dr L Li, Dr C Lovelock, and Dr W Kuker collected data and revised the manuscript. Dr M Liu provided study design and supervision. Dr PM Rothwell conceived and designed the overall study, provided study supervision and funding, acquired, analyzed, and interpreted data, and wrote and revised the manuscript.

## Sources of Funding

The Oxford Vascular Study is funded by the National Institute for Health Research (NIHR), Oxford Biomedical Research Centre (BRC), Wellcome Trust, Wolfson Foundation, British Heart Foundation, and the European Union’s Horizon 2020 research and innovation programme under grant agreement 666881, SVDs@target. Professor Rothwell is in receipt of an NIHR Senior Investigator award. Dr Liu is funded by the China Scholarship Council. The views expressed are those of the author(s) and not necessarily those of the NHS, the NIHR, or the Department of Health.

## Disclosures

None.

## Supplementary Material

## References

[1] O’Rourke MF, Safar ME (2005). Relationship between aortic stiffening and microvascular disease in brain and kidney: cause and logic of therapy.. Hypertension.

[2] Seliger SL, Longstreth WT (2008). Lessons about brain vascular disease from another pulsating organ, the kidney.. Stroke.

[3] Ryu WS, Lee SH, Kim CK, Kim BJ, Yoon BW (2012). The relation between chronic kidney disease and cerebral microbleeds: difference between patients with and without diabetes.. Int J Stroke.

[4] Umemura T, Kawamura T, Sakakibara T, Mashita S, Hotta N, Sobue G (2012). Microalbuminuria is independently associated with deep or infratentorial brain microbleeds in hypertensive adults.. Am J Hypertens.

[5] Ovbiagele B, Wing JJ, Menon RS, Burgess RE, Gibbons MC, Sobotka I (2013). Association of chronic kidney disease with cerebral microbleeds in patients with primary intracerebral hemorrhage.. Stroke.

[6] Akoudad S, Sedaghat S, Hofman A, Koudstaal PJ, van der Lugt A, Ikram MA (2015). Kidney function and cerebral small vessel disease in the general population.. Int J Stroke.

[7] Xiao L, Lan W, Sun W, Dai Q, Xiong Y, Li L (2015). Chronic kidney disease in patients with lacunar stroke: association with enlarged perivascular spaces and total magnetic resonance imaging burden of cerebral small vessel disease.. Stroke.

[8] Makin SD, Cook FA, Dennis MS, Wardlaw JM (2015). Cerebral small vessel disease and renal function: systematic review and meta-analysis.. Cerebrovasc Dis.

[9] Staals J, Makin SD, Doubal FN, Dennis MS, Wardlaw JM (2014). Stroke subtype, vascular risk factors, and total MRI brain small-vessel disease burden.. Neurology.

[10] Klarenbeek P, van Oostenbrugge RJ, Rouhl RP, Knottnerus IL, Staals J (2013). Ambulatory blood pressure in patients with lacunar stroke: association with total MRI burden of cerebral small vessel disease.. Stroke.

[11] Li L, Yiin GS, Geraghty OC, Schulz UG, Kuker W, Mehta Z, Oxford Vascular Study (2015). Incidence, outcome, risk factors, and long-term prognosis of cryptogenic transient ischaemic attack and ischaemic stroke: a population-based study.. Lancet Neurol.

[12] Lau KK, Li L, Schulz U, Simoni M, Chan KH, Ho SL (2017). Total small vessel disease score and risk of recurrent stroke: validation in 2 large cohorts.. Neurology.

[13] Kasiske BL, Wheeler DC (2013). Kdigo clinical practice guideline for the evaluation and management of chronic kidney disease foreword.. Kidney Int Suppl.

[14] Wardlaw JM, Smith EE, Biessels GJ, Cordonnier C, Fazekas F, Frayne R, STandards for ReportIng Vascular changes on nEuroimaging (STRIVE v1) (2013). Neuroimaging standards for research into small vessel disease and its contribution to ageing and neurodegeneration.. Lancet Neurol.

[15] Greenberg SM, Vernooij MW, Cordonnier C, Viswanathan A, Al-Shahi Salman R, Warach S, Microbleed Study Group (2009). Cerebral microbleeds: a guide to detection and interpretation.. Lancet Neurol.

[16] Potter GM, Chappell FM, Morris Z, Wardlaw JM (2015). Cerebral perivascular spaces visible on magnetic resonance imaging: development of a qualitative rating scale and its observer reliability.. Cerebrovasc Dis.

[17] Fazekas F, Chawluk JB, Alavi A, Hurtig HI, Zimmerman RA (1987). MR signal abnormalities at 1.5 T in Alzheimer’s dementia and normal aging.. AJR Am J Roentgenol.

[18] Ovbiagele B, Liebeskind DS, Pineda S, Saver JL (2010). Strong independent correlation of proteinuria with cerebral microbleeds in patients with stroke and transient ischemic attack.. Arch Neurol.

[19] Thompson CS, Hakim AM (2009). Living beyond our physiological means: small vessel disease of the brain is an expression of a systemic failure in arteriolar function: a unifying hypothesis.. Stroke.

[20] Akhurst RJ, Hata A (2012). Targeting the TGFβ signalling pathway in disease.. Nat Rev Drug Discov.

[21] Akoudad S, Ikram MA, Koudstaal PJ, Hofman A, van der Lugt A, Vernooij MW (2013). Cerebral microbleeds and the risk of mortality in the general population.. Eur J Epidemiol.

[22] Levey AS, Bosch JP, Lewis JB, Greene T, Rogers N, Roth D (1999). A more accurate method to estimate glomerular filtration rate from serum creatinine: a new prediction equation. Modification of Diet in Renal Disease Study Group.. Ann Intern Med.

