# Supplementary material for: Age-Specific Associations of Renal Impairment With Magnetic Resonance Imaging Markers of Cerebral Small Vessel Disease in Transient Ischemic Attack and Stroke
Source: Stroke. 2018 Mar 26;49(4):899–904. doi: 10.1161/STROKEAHA.117.019650 (PMC5895118; doi:10.1161/STROKEAHA.117.019650)
Supplement: Supplementary file 1 [file str-49-899-s001.pdf]

SUPPLEMENTAL MATERIAL

Supplemental Method. OXVASC methodology

Study population

The Oxford Vascular Study (OXVASC) is a prospective, population-based cohort study of all incident acute vascular events in all territories (transient ischaemic attack, stroke, acute coronary and peripheral vascular events).

The study population consisted of all 92,728 individuals, irrespective of age, registered with 100 general practitioners (GPs) in nine general practices in Oxfordshire, UK. In the UK, general practices provide primary health care for registered individuals and hold a lifelong record of all medical consultations (from the National Health Service [NHS] and private health care), and details of treatments, blood pressure, and investigations. In Oxfordshire, an estimated 97% of the true residential population is registered with a general practice, with most non-registered individuals being young adults. All participating practices held accurate age-sex patient registers, and allowed regular searches of their computerised diagnostic coding systems. The practices had all collaborated on a previous population-based study, for which they were originally selected to be representative of the urban and rural mix and the deprivation range of Oxfordshire as a whole.[1] Based on the index of multiple deprivation (IMD), the population was less deprived than the rest of England, but had a broad range of deprivation.

The OXVASC population is 94% white people, 3% Asian, 2% Chinese, and 1% Afro-Caribbean.[2] The proportion of whites is similar to that of the UK as a whole (88% white) and to many other western countries (Australia - 90%; France - 91%; Germany - 93.9%).

Case ascertainment

After a 3-month pilot study, the study started on April 1, 2002, and is ongoing. Ascertainment combined prospective daily searches for acute events (hot pursuit) and retrospective searches of hospital-care and primary-care administrative and diagnostic coding data (cold pursuit).

Hot pursuit was based on:

1) A daily (weekdays only), urgent open-access "TIA clinic" to which participating general practitioners (GPs) and the local accident and emergency department (A&E) send all individuals with suspected TIA or stroke whom they would not normally admit to hospital, with alternative on-call review provision at weekends. Patients too frail to attend are assessed at their residence by a study nurse or doctor.
2) Daily searches and case note review of admissions to the Emergency Assessment Unit, Medical Short Stay Unit, Coronary Care Unit and Cardiothoracic Critical Care Unit, Cardiology, Cardiothoracic, and Vascular Surgery wards, Acute Stroke Unit, Neurology ward and all other general wards when indicated.
3) Daily searches of the local A&E and eye hospital attendance registers.
4) Daily identification via the Bereavement Office of patients dead on arrival at hospital or who died soon after.
5) Daily searches of lists of all patients from the study population in whom a troponin-I level had been requested.

6) Daily assessment of all patients undergoing diagnostic coronary, carotid and peripheral angiography, angioplasty, stenting or vascular surgical procedures in any territory to identify both total burden of vascular invention and any potential missed prior acute events.

Cold pursuit procedures were:

1) Frequent visits to the study practices and monthly searches of practice diagnostic codes.
2) Monthly practice-specific list of all patients admitted to all acute and community NHS hospitals.
3) Monthly listings of all referrals for brain or carotid imaging studies performed in local hospitals.
4) Monthly reviews of all death certificates and coroners reports to review out-of-hospital deaths.
5) Practice-specific listings of all ICD-10 death codes from the local Department of Public Health.

Patients found on GP practice searches who have an event whilst temporarily out of Oxfordshire are included, but visitors who were not registered with one of the study practices are excluded. A study clinician assessed patients as soon as possible after the event in the hospital or at home. Informed consent was sought, if possible, or assent was obtained from a relative.

Data is collected using event-specific forms, for TIA and stroke, acute coronary syndrome or acute peripheral vascular events. Standardised clinical history and cardiovascular examination are recorded. Information recorded from the patient, their hospital records and their general practice records includes details of the clinical event, medication, past medical history, all investigations relevant to their admission (including blood results, electrocardiography, brain imaging and vascular imaging-duplex ultrasonography, CT-angiography, MR-angiography or DSA) and all interventions occurring subsequent to the event.

If a patient died before assessment, we obtained an eyewitness account of the clinical event and reviewed any relevant records. If death occurred outside the hospital or before investigation, the autopsy result was reviewed. Clinical details are sought from primary care physicians or other clinicians on all deaths of possible vascular aetiology.

All surviving patients are followed-up face-to-face at 1, 6, 12, 60 and 120 months after the initial event by a research nurse or physician and all recurrent vascular events were recorded together with the relevant clinical details and investigations. If face-to-face follow up is not possible, telephone follow-up is performed or enabled via the general practitioner. All recurrent vascular events that presented to medical attention would also be identified acutely by ongoing daily case ascertainment within OXVASC. If a recurrent vascular event was suspected at a follow-up visit or referred by the GPs to clinic or admitted, the patient was re-assessed and investigated by a study physician.

Definition of diagnosis

Although new definitions for stroke and TIA have been suggested recently,[2,3] in order to enable comparison with previous studies, the classic definitions of TIA and stroke are used throughout.[4] A stroke is defined as rapidly developing clinical symptoms and/or signs of focal, and at time global (applied to patients in deep coma and to those with subarachnoid haemorrhage), loss of brain function, with symptoms lasting more than 24 hours or leading to death, with no apparent cause other than that of vascular origin.[4] A TIA is an acute loss of focal brain

or monocular function with symptoms lasting less than 24 hours and which is thought to be caused by inadequate cerebral or ocular blood supply as a result of arterial thrombosis, low flow or embolism associated with arterial, cardiac or haematological disease.[4] All diagnoses were reviewed by a senior neurologist (PMR). With the high rate (97%) of imaging or autopsy in OXVASC, strokes of unknown type were coded as ischaemic.

Brain imaging protocol

From April 1, 2002, to March 31, 2010 (phase 1), MRI and magnetic resonance angiography (MRA) was performed in selected patients when clinically indicated. From April 1, 2010 onwards (phase 2), brain MRI and MRA became the first-line imaging methods.[5] Patients were scanned predominantly with 2 scanners: Achieva (Philips Healthcare, Best, the Netherlands) and Magnetom Verio (Siemens health care, Munich, Germany).[6] The detailed sequence parameters are listed in the table below. One neuroradiologists (W.K.) provided ongoing supervision of interpretation of the MRI throughout the study period. The intra-rater κ for 50 randomly selected scans was as follows: lacunes 0.85; microbleed burden (0, 1, 2–4, ≥5) 0.88; periventricular WMH burden (Fazekas grade 0, 1, 2, 3) 0.66; subcortical WMH burden (Fazekas grade 0, 1, 2, 3) 0.75; PVS burden (<11, 11–20, >20) 0.86 (BG), 0.84 (CS).[7]

Table. Imaging sequence parameters used in OXVASC

MR parameters	OXVASC scanner 1 Magnetom Verio, Siemens Healthcare	OXVASC scanner 2 Discovery MR750, GE Healthcare	OXVASC scanner 3 Achieva, Philips Healthcare	OXVASC scanner 4 Signa HDxt, GE Healthcare
**Patients scanned**	388	62	493	137
**Field strength (T)**	3	3	1.5	1.5
**T1W TR/TE/TI (ms)**	2000/1.94/880	-	701/16	-
**T2W TR/TE (ms)**	6000/96	5800/94	5061/100	3760/100
**FLAIR TR/TE/TI (ms) (3D)**	9000/88/2500	9600/130/2350	11000/140/2800	8080/112/2200
**Diffusion TR/TE (ms)**	5300/91	6000/84	2891/73	6100/71
**GRE / SWI TR/TE (ms) (3D)**	GRE 504/15	GRE 500/20	GRE 694/23	GRE 560/25
**Pixel bandwidth (Hz)**	240 (T1W)<br>220 (T2W)<br>202 (FLAIR)<br>1374 (Diffusion)<br>200 (GRE)	-<br>50 (T2W)<br>41.7 (FLAIR)<br>250 (Diffusion)<br>31.3 (GRE)	87.4 (T1W)<br>88.5 (T2W)<br>375 (FLAIR)<br>25.3 (Diffusion)<br>109.3 (GRE)	-<br>47.6 (T2W)<br>31.3 (FLAIR)<br>-<br>75 (GRE)
**Matrix**	256x256 (T1W)<br>320x320 (T2W)<br>192x192 (FLAIR)<br>130x130 (Diffusion)<br>320x256 (GRE)	-<br>512 (T2W)<br>384x224 (FLAIR)<br>128x128 (Diffusion)<br>288x224 (GRE)	118x214 (T1W)<br>356x193 (T2W)<br>236x159 (FLAIR)<br>97x84 (Diffusion)<br>256x163 (GRE)	416x256 (T2W)<br>256x224 (FLAIR)<br>128x128 (Diffusion)<br>288x192 (GRE)
**No. of slices**	208 (T1W)<br>25 (T2W)<br>50 (FLAIR)<br>25 (Diffusion)<br>25 (GRE)	25	25 (T1W)<br>25 (T2W)<br>28 (FLAIR)<br>25 (Diffusion)<br>22 (GRE)	25
**Slice thickness (mm)**	1 (T1W)<br>5 (T2W)<br>3 (FLAIR)<br>5 (Diffusion)<br>5 (GRE)	5	5	5
**Inter-slice gap (mm)**	0 (T1W)<br>1 (T2W)<br>0 (FLAIR coronal)<br>1 (Diffusion)<br>1 (GRE)	1	1	1
**Voxel size (mm^3)**	1.0x1.0x1.0 (T1W)<br>0.8x0.8x5.0 (T2W)<br>1.0x1.0x3.0 (FLAIR)<br>1.8x1.8x5.0 (Diffusion)<br>0.9x0.8x5.0 (GRE)	-	0.53x0.53x5.0 (T1W)<br>0.65x0.65x5.0 (T2W)<br>0.82x0.81x5.0 (FLAIR)<br>1.74x1.73x5.0 (Diffusion)<br>0.90x0.90x5.0 (GRE)	-

References

1. Bamford J, Sandercock P, Dennis M, Burn J, Warlow C. A prospective study of acute cerebrovascular disease in the community: the Oxfordshire Community Stroke Project--1981-86. 2. Incidence, case fatality rates and overall outcome at one year of cerebral infarction, primary intracerebral and subarachnoid haemorrhage. *J Neurol Neurosurg Psychiatry* 1990;53:16-22.
2. Easton JD, Saver JL, Albers GW, et al. Definition and evaluation of transient ischemic attack: a scientific statement for healthcare professionals from the American Heart Association/American Stroke Association Stroke Council; Council on Cardiovascular Surgery and Anesthesia; Council on Cardiovascular Radiology and Intervention; Council on Cardiovascular Nursing; and the Interdisciplinary Council on Peripheral Vascular Disease. The American Academy of Neurology affirms the value of this statement as an educational tool for neurologists. *Stroke* 2009;40:2276-2293.
3. Sacco RL, Kasner SE, Broderick JP, et al. An updated definition of stroke for the 21st century: a statement for healthcare professionals from the American Heart Association/American Stroke Association. *Stroke* 2013;44:2064-2089.
4. Hatano S. Experience from a multicentre stroke register: a preliminary report. *Bulletin of the World Health Organization* 1976;54:541-553.
5. Li L, Yiin GS, Geraghty OC, et al. Incidence, outcome, risk factors, and long-term prognosis of cryptogenic transient ischaemic attack and ischaemic stroke: a population-based study. *Lancet Neurol* 2015;14:903-913.
6. Simoni M, Li L, Paul NL, et al. Age- and sex-specific rates of leukoaraiosis in TIA and stroke patients: population-based study. *Neurology* 2012;79:1215-1222.
7. Lau KK, Li L, Schulz U. Total small vessel disease score and risk of recurrent stroke: validation in 2 large cohorts. *Neurology* 2017;88:2260-2267.

Supplemental Table I. Baseline charateristics of patient with renal impairment vs. patients without renal impairment

	All	Patients with renal impairment	Patients without renal impairment	p
	**(N=1028)**	**(n=300)**	**(n=728)**	
Age (mean±SD; years)	68.4±14.1	76.7±9.9	65.0±14.1	<0.0001
Female	490 (47.7)	172 (57.3)	318 (43.7)	<0.0001
Hypertension	563 (54.8)	223 (74.3)	340 (46.7)	<0.0001
Hyperlipidemia	381 (37.1)	156 (52.0)	225 (30.9)	<0.0001
Diabetic mellitus	136 (13.2)	57 (19.0)	79 (10.9)	0.0005
Ever smoker	521 (50.7)	149 (49.7)	372 (51.2)	0.66
Atrial fibrillation	160 (15.6)	63 (21.0)	97 (13.3)	0.002
TIA/stroke prior to the index event	187 (18.2)	91 (30.3)	96 (13.2)	<0.0001
History of ischaemic heart disease	141 (13.7)	67 (22.3)	74 (10.2)	<0.0001
Type of index event – ischaemic stroke	486 (47.3)	151 (50.3)	335 (46.0)	0.21
Aetiology by TOAST classification				0.003
Large artery disease	137 (13.3)	58 (19.3)	102 (14.0)	
Cardioembolic	160 (15.6)	52 (17.3)	85 (11.7)	
Small vessel disease	124 (12.1)	31 (10.3)	93 (12.8)	
Cryptogenic	514 (50.0)	131 (43.7)	383 (52.6)	
Unknown aetiology	26 (2.5)	8 (2.7)	18 (2.5)	
Multiple aetiology	35 (3.4)	15 (5.0)	20 (2.7)	
Other aetiology	32 (3.1)	5 (1.7)	27 (3.7)	
SVD score				<0.0001
0	387 (37.6)	75 (25.0)	312 (42.9)	
1	293 (28.5)	88 (29.3)	205 (28.2)	
2	215 (20.9)	77 (25.7)	138 (19.0)	
≥3	133 (12.9)	60 (20.0)	73 (10.0)	
BP at index event (mean/SD)				
Systolic blood pressure (mmHg)	150.1±24.4	151.8±27.3	149.4±23.1	0.23
Diastolic blood pressure (mmHg)	83.8±13.2	80.7±13.3	85.1±13.0	<0.0001
All BP prior to the event (mean/SD)				
Systolic blood pressure (mmHg)	138.7±14.3	144.5±13.0	136.2±14.1	<0.0001
Diastolic blood pressure (mmHg)	80.0±7.7	80.0±7.0	80.1±7.9	0.84

*renal impairment is defined as eGFR<60 mL/min/1.73m^2

Supplemental Table II. Associations of renal impairment and total SVD score stratified by age in Lacunar vs. Non-lacunar events

	Lacunar						Non-lacunar					
	Crude		Model I*		Model II**		Crude		Model I*		Model II**	
	OR (95%CI)	p	OR1 (95%CI)	p1	OR2 (95%CI)	p2	OR (95%CI)	p	OR1 (95%CI)	p1	OR2 (95%CI)	p2
**Overall**	1.55 (0.75-3.20)	0.235	0.80 (0.37-1.73)	0.572	0.79 (0.35-1.80)	0.575	2.32 (1.78-3.01)	<0.001	0.95 (0.71-1.27)	0.727	0.74 (0.53-1.02)	0.066
**Stratified by age**												
<60y	16.04 (2.28-112.62)	0.005	21.28 (2.38-190.57)	0.006	14.01(1.31-149.45)	0.029	2.22 (0.78-6.32)	0.134	1.44 (0.49-4.25)	0.508	1.87 (0.59-5.91)	0.285
60-79y	0.48 (0.18-1.30)	0.150	0.44 (0.16-1.22)	0.114	0.46 (0.15-1.40)	0.171	1.15 (0.80-1.65)	0.441	0.98 (0.67-1.42)	0.903	0.73 (0.48-1.12)	0.148
≥80y	0.88 (0.17-4.70)	0.885	0.70 (0.13-3.87)	0.681	0.87 (0.11-6.56)	0.889	0.96 (0.58-1.59)	0.867	0.93 (0.56-1.54)	0.780	0.69 (0.38-1.25)	0.219

SVD=small vessel disease, OR=odds ratio, CI=confidence interval; *Model I: adjusted for age and gender; **Model II: adjusted for age, gender, history of hypertension, diabetes and premorbid mean systolic blood pressure.

Renal impairment is defined as eGFR<60 mL/min/1.73m^2

Supplemental Table III. Associations of renal impairment and the presence of individual small vessel disease markers stratified by age and adjusted for age/sex and for known vascular risk factors

	Renal impairment in those with SVD (n/total; %)	Renal impairment in those without SVD (n/total; %)	p	Model I* OR1	95%CI	p1	Model II** OR2	95% CI	p2
**Cerebral microbleeds**									
<60y	5/16 (31.3)	16/245 (6.5)	**0.001**	5.01	1.48-16.94	**0.010**	5.84	1.45-23.53	**0.013**
60-79y	30/83 (36.1)	125/466 (26.8)	**<0.001**	1.31	0.90-1.90	0.157	1.19	0.66-2.14	0.557
≥80y	32/57(56.1)	92/161 (57.1)	0.963	0.99	0.54-1.83	0.988	0.89	0.43-1.84	0.742
**Moderate-severe periventricular WMH**									
<60y	5/15 (33.3)	16/246 (6.5)	**<0.001**	5.62	1.90-16.73	**0.002**	6.28	1.54-25.63	**0.010**
60-79y	47/168 (28.0)	108/381 (28.3)	**<0.001**	0.92	0.62-1.38	0.696	0.65	0.40-1.05	0.079
≥80y	61/114 (53.5)	63/104 (60.6)	0.689	0.70	0.41-1.20	0.196	0.68	0.36-1.26	0.219
**Moderate-severe subcortical WMH**									
<60y	3/17 (17.6)	18/244 (7.4)	**0.031**	2.07	0.67-6.45	0.212	1.41	0.32-6.12	0.648
60-79y	48/164 (29.3)	107/385 (27.8)	0.300	1.02	0.68-1.52	0.925	0.79	0.49-1.29	0.346
≥80y	61/113 (54.0)	63/105 (60.0)	0.194	0.68	0.40-1.16	0.154	0.57	0.30-1.08	**0.084**
**Moderate-severe basal ganglia PVS(>10)**									
<60y	5/33 (15.2)	16/228 (7.0)	0.108	1.53	0.50-4.68	0.459	1.62	0.50-5.31	0.425
60-79y	74/300 (24.7)	81/249 (32.5)	**0.042**	0.56	0.37-0.83	**0.004**	0.45	0.29-0.71	**0.001**
≥80y	94/168 (56.0)	30/50 (60.0)	0.612	0.80	0.42-1.54	0.500	0.62	0.29-1.35	0.231
**Lacunes**									
<60y	7/29 (24.1)	14/232 (6.0)	**0.004**	3.42	1.20-9.75	0.022	3.81	1.21-12.04	**0.023**
60-79y	34/100 (34.0)	121/449 (26.9)	0.177	1.37	0.85-2.20	0.202	1.19	0.70-2.05	0.520
≥80y	35/53 (66.0)	89/165 (53.9)	0.151	1.66	0.87-3.18	0.125	1.08	0.52-2.22	0.836

SVD=small vessel disease, WMH=white matter hyperintensity, PVS= perivascular spaces, OR=odds ratio, CI=confidence interval; *Model I: adjusted for age, and gender; **Model II: adjusted for age, gender, history of hypertension, diabetes and premorbid mean systolic blood pressure. Renal impairment is defined as eGFR<60 mL/min/1.73m^2

Supplemental Table IV. Associations of renal impairment and total SVD score by age, using creatinine taken one year prior to the index event

	Crude			Model I*			Model II**		
	**OR**	**95%CI**	**p**	**OR1**	**95%CI**	**p1**	**OR2**	**95% CI**	**p2**
**Overall**	1.65	1.25-2.19	<0.001	0.87	0.64-1.19	0.377	0.73	0.52-1.04	0.079
**Stratified by age**									
<60 years	4.40	1.44-13.45	0.009	3.52	1.11-11.21	0.033	2.95	0.82-10.55	0.096
60-79 years	1.09	0.74-1.61	0.655	0.92	0.62-1.39	0.704	0.89	0.56-1.39	0.595
≥80 years	0.65	0.39-1.10	0.108	0.66	0.39-1.12	0.128	0.42	0.23-0.78	0.006

SVD=small vessel disease, OR=odds ratio, CI=confidence interval; *Model I: adjusted for age, gender; **Model II: adjusted for age, gender, history of hypertension, diabetes and premorbid mean systolic blood pressure. The first two groups of the total SVD (score 0 and 1) were combined for the ordinal regression. Renal impairment is defined as eGFR<60 mL/min/1.73m^2

Supplemental Table V. Associations of renal impairment and the presence of individual small vessel disease marker by age, using creatinine taken one year prior to the index event

	Renal impairment in those with SVD	Renal impairment in those without SVD		Model I*			Model II**		
	(n/total; %)	(n/total; %)	p	OR1	95%CI	p1	OR2	95% CI	p2
**Cerebral microbleeds**									
<60y	3/12 (25.0)	19/152 (12.5)	0.205	1.92	0.45-8.24	0.380	2.00	0.43-9.43	0.380
60-79y	27/70 (38.6)	130/403 (32.3)	0.336	1.19	0.69-2.04	0.540	0.91	0.50-1.68	0.772
≥80y	30/56 (53.6)	97/147 (66.0)	0.108	0.63	0.34-1.18	0.150	0.59	0.28-1.23	0.158
**Moderate-severe periventricular WMH**									
<60y	4/14 (28.6)	18/150 (12.0)	0.098	2.22	0.67-7.33	0.193	2.46	0.58-10.44	0.221
60-79y	53/150 (37.3)	104/323 (32.2)	0.530	1.00	0.66-1.52	0.992	0.85	0.53-1.35	0.486
≥80y	63/108 (58.3)	64/95 (67.4)	0.194	0.68	0.39-1.21	0.189	0.69	0.36-1.31	0.254
**Moderate-severe subcortical WMH**									
<60y	3/13 (23.1)	19/151 (12.6)	0.386	1.43	0.40-5.08	0.583	1.67	0.40-6.94	0.483
60-79y	45/141 (31.9)	112/332 (33.7)	0.749	0.80	0.53-1.23	0.318	0.70	0.43-1.13	0.145
≥80y	66/109 (60.6)	61/94 (64.9)	0.563	0.82	0.47-1.46	0.504	0.62	0.31-1.23	0.172
**Moderate-severe basal ganglia PVS(>10)**									
<60y	4/22 (18.2)	18/142 (12.7)	0.502	1.19	0.35-4.03	0.785	1.07	0.29-3.95	0.920
60-79y	78/253 (30.8)	79/220 (35.9)	0.282	0.61	0.41-0.93	0.020	0.60	0.38-0.95	0.029
≥80y	96/155 (61.9)	31/48 (64.6)	0.865	0.92	0.46-1.82	0.800	0.77	0.33-1.76	0.531
**Lacunes**									
<60y	5/24 (20.8)	17/140 (12.1)	0.502	1.09	1.01-1.17	0.472	1.39	0.40-4.77	0.606
60-79y	36/88 (40.9)	121/385 (31.4)	0.282	0.90	0.56-1.44	0.666	1.62	0.94-2.82	0.085
≥80y	31/49 (63.3)	96/154 (62.3)	0.865	1.03	0.53-2.02	0.915	0.63	0.30-1.33	0.222

SVD=small vessel disease, WMH=white matter hyperintensity, PVS= perivascular spaces, OR=odds ratio, CI=confidence interval; *Model I: adjusted for age, gender; **Model II: adjusted for age, gender, history of hypertension, diabetes and premorbid mean systolic blood pressure. . Renal impairment is defined as eGFR<60 mL/min/1.73m^2
